# Role of Publicly-Funded Molecular Testing in Surgical Management of Thyroid Nodules within Canadian Medicare: Clinical Assessment of ThyroSeqv3 Molecular Test Pilot Project at McGill University

**DOI:** 10.1177/19160216251336687

**Published:** 2025-05-28

**Authors:** Saruchi Bandargal, Jessica Hier, Mawaddah Abdulhaleem, Véronique-Isabelle Forest, Maryse Brassard, Geneviève Rondeau, Orr Dimitstein, Marco A. Mascarella, Alex Mlynarek, Michael P. Hier, Keith Richardson, Nader Sadeghi, Karen M. Kost, Anthony Zeitouni, Marc Philippe Pusztaszeri, Pierre Fortier, Danielle Beaudoin, Marie-Helene Massicotte, Andree Boucher, Richard J. Payne

**Affiliations:** 1 Faculty of Medicine, McGill University, Montreal, QC, Canada; 2 Department of Otolaryngology—Head and Neck Surgery, McGill University, Montreal, QC, Canada; 3 Department of Otolaryngology—Head and Neck Surgery, Jewish General Hospital, McGill University, Montreal, QC, Canada; 4 Department of Otolaryngology—Head and Neck Surgery, Royal Victoria Hospital, McGill University, Montreal, QC, Canada; 5 Division of Endocrinology, Department of Medicine, Université Laval, Centre Hospitalier Universitaire de Québec, Quebec City, QC, Canada; 6 Division of Endocrinology, Department of Medicine, Université de Montréal, Centre Hospitalier de l’Université de Montréal, Montreal, QC, Canada; 7 Department of Pathology, Jewish General Hospital, McGill University, Montreal, QC, Canada; 8 Department of Otolaryngology—Head and Neck Surgery, Université de Sherbrooke, Sherbrooke, QC, Canada; 9 Department of Otolaryngology—Head and Neck Surgery, Université Laval, Centre Hospitalier Universitaire de Québec, Quebec City, QC, Canada

**Keywords:** Thyroseqv3, molecular testing, Canadian Medicare, indeterminate thyroid nodule, surgical management

## Abstract

**Importance:**

Recently, the Québec public health care system established a pilot project to cover costs of molecular testing for select patients with cytologically-indeterminate thyroid nodules.

**Objective:**

This study aimed to evaluate the clinical utility of the ThyroSeqv3 molecular test pilot project at McGill University in surgical management of thyroid nodules within Canada’s single-payer health care system.

**Design:**

Multicenter cohort study, in liaison with the Québec Health Ministry.

**Setting:**

Jewish General Hospital and Royal Victoria Hospital in Montreal, Canada.

**Participants:**

Patients with a Bethesda III or IV and TIRADS 3 or 4 thyroid nodule measuring between 1 and 4 cm in size on ultrasound were analyzed across pre- and post-pilot project phases.

**Intervention:**

The pre-pilot project surgical control group included patients who underwent surgical intervention, excluding those who opted for out-of-pocket molecular testing. The post-pilot project surgical exposure group encompassed participants in the pilot project, undergoing publicly-funded ThyroSeqv3 molecular testing and subsequent surgical intervention.

**Main Outcome Measures:**

Surgical malignancy/noninvasive follicular thyroid neoplasm with papillary-like nuclear features (NIFTP) rate.

**Results:**

A total of 314 patients qualified for the pilot project, with 207 (65.9%) having Bethesda III nodules and 107 (34.1%) having Bethesda IV nodules. Molecular testing yielded a result of negative in 238 (75.8%) cases and positive in 76 (24.2%) cases. Histopathology reports of positive patients who opted for surgery revealed a surgical malignancy/NIFTP rate of 73.1%. The surgical malignancy/NIFTP rate at our institution prior to the implementation of the pilot project for patients adhering to the inclusion criteria was statistically significantly lower at 47.9% (*P* = .0025).

**Conclusions:**

The ThyroSeqv3 molecular test pilot project has improved upon physicians’ traditional clinical practice by enabling a wider patient population to access this otherwise costly technology. It not only curtailed futile diagnostic hemithyroidectomies but also led to a more discerning allocation of surgeries, as corroborated by an increased surgical malignancy/NIFTP rate post-implementation.

**Relevance:**

The results of our study suggest that publicly-funded molecular testing could contribute positively to the Canadian single-payer health care system by optimizing patient outcomes as well as fiscal policy.

## Graphical abstract

**Graphical Abstract:**
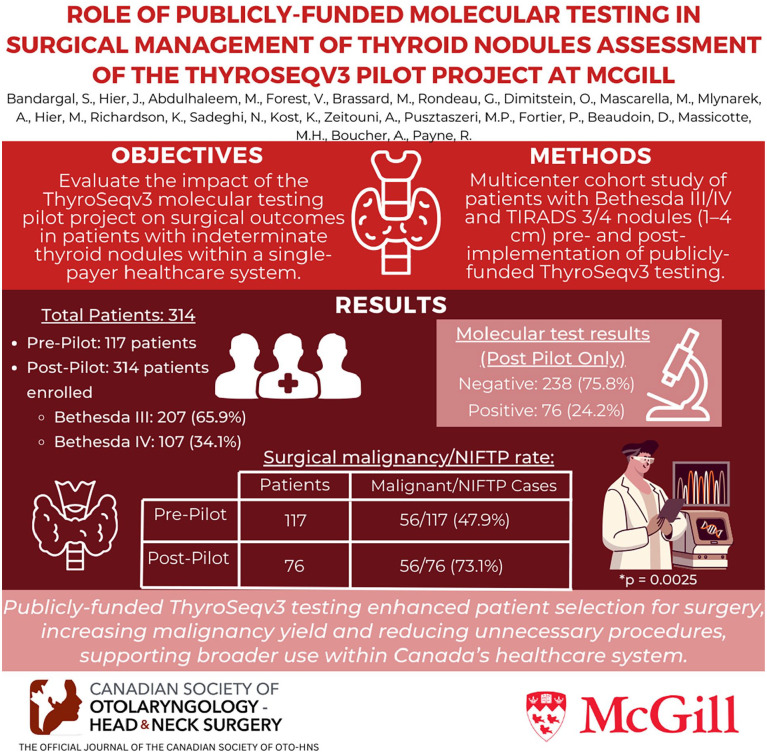

## Key Message

• The ThyroSeqv3 pilot project improved patient selection, increasing the surgical malignancy/noninvasive follicular thyroid neoplasm with papillary-like nuclear features rate.
• Expanded access curtailed futile diagnostic hemithyroidectomies, optimizing intervention.

## Introduction

Thyroid cancer imposes a considerable weight on Canada’s health care system, with increasing incidence rates on account of enhanced diagnostic capabilities of modern medical technology and rising health care expenditures.^1,2^ According to the Canadian Cancer Society, it is one of the fastest growing cancers in Canada, encompassing a 5% tumor-based prevalence of all cancers over a 25 year period.^3^ The diagnosis of and treatment for thyroid cancer necessitate a comprehensive care paradigm that involves an array of medical professionals, including primary care physicians, endocrinologists, radiologists, pathologists, and surgeons.^4-6^ This multidisciplinary approach can result in increased health care utilization, including diagnostic testing, surgical procedures, and postoperative lifelong follow-up.

The main hindrance encountered in the diagnosis of thyroid cancer is the high rate of inconclusive results obtained from fine needle aspiration (FNA) biopsy, the gold standard for examining thyroid nodules. Twenty percent to 30% of FNA biopsies can be indeterminate, yielding a diagnosis of Bethesda III or IV.^7,8^ In such instances, clinicians often recommend diagnostic surgery as the next viable option, which carries with it a significant expense, risk of complications, long waitlists, cosmetic concerns, psychological impact, and futility in patients with benign disease. Beyond the immediate expenditures associated with surgery, indirect costs may also arise, including the loss of productivity and wages for patients who require time off from work to recuperate following the operation.^9^

Molecular testing of thyroid nodules can provide important diagnostic and prognostic insights, guide treatment decisions, and obviate diagnostic surgery.^10-12^ Advances in this nascent technology, particularly with the advent of the ThyroSeqv3 molecular test, have appreciably improved the accuracy of thyroid nodule diagnosis and bridged the knowledge gap concerning indeterminate results.^11^ Nonetheless, on account of its novelty, molecular tests can be quite expensive, imposing a formidable hurdle for many patients in accessing optimal treatment.^13^ In an attempt to circumvent this financial barrier for many patients, and better public health and fiscal policy, Québec’s Health Ministry initiated a pilot project that publicly funds molecular testing for select patients with cytologically-indeterminate thyroid nodules (CITNs) beginning November 2021. Our study’s aim was to assess the clinical utility of the novel ThyroSeqv3 molecular test pilot project in surgical management of thyroid nodules, considering the single-payer health care system in Canada.

## Materials and Methods

### Study Design

This multicenter cohort study, spanning 2021 to 2023, was conducted at the Jewish General Hospital and the Royal Victoria Hospital in Montreal, Canada, under approval from the Medical-Bioethics Research Ethics Committee of the Integrated Health and Social Services Network for West-Central Montreal (MP-05-2023-3746), in accordance with the 2013 Declaration of Helsinki. Patient data encompassed demographics, preoperative FNA biopsies, molecular testing, when available, and postoperative pathology. Consent for the pilot project was obtained through a general written consent form utilized across our institutions.

The ThyroSeqv3 molecular test pilot project, a joint venture launched by the Québec Health Ministry in concert with the province’s 4 academic institutions, facilitates coverage of ThyroSeqv3 molecular testing expenses for patients diagnosed with thyroid nodules that are 1 to 4 cm in size, classified as Bethesda III (on 2 separate FNA biopsies) or Bethesda IV, and TIRADS 3 or 4 who did not have another indication for surgery (Graves’ disease, multinodular goiters, compressive symptoms, etc).

### Patient Population

The study’s patient population was systematically defined to allow for a meaningful comparative analysis between pre- and post-pilot project phases, and as such, the inclusion criteria for both phases were restricted to the aforementioned pilot project’s eligibility requirements.

The pre-pilot project phase comprised individuals who underwent surgery between May 2021 and March 2022, excluding those who opted for molecular testing, an out-of-pocket expense at the time as these data in their entirety was not possible to obtain. This cohort served as the surgical control group for our study, providing a realistic baseline for surgical malignancy/noninvasive follicular thyroid neoplasm with papillary-like nuclear features (NIFTP) rates before the introduction of publicly-funded molecular testing.

The post-pilot project phase included those who were part of the pilot project, whether they ultimately underwent surgery (between April 2022 and February 2023) or not. These patients, comprising our surgical exposure group, underwent ThyroSeqv3 molecular testing as part of the pilot project’s provisions.

### Tumor Analysis

FNA specimens, collected with 21 to 25 G needles under ultrasound guidance, were transported to the University of Pittsburgh Medical Center for ThyroSeqv3 analysis. Surgical-resected specimens were examined by subspecialized pathologists for aggressive features including macroscopic extrathyroidal extension, lymph node metastasis, poorly-differentiated thyroid carcinoma, and histological features indicating a high risk such as tall cell, solid/trabecular, columnar cell, micropapillary/hobnail, and diffuse sclerosing subtypes. Furthermore, all tumors were classified in keeping with the latest WHO 2022 classification of thyroid tumors.^14^

### Statistical Analysis

Data were analyzed using the SAS Studio 3.8 software. Variables such as age, sex, Bethesda score, preoperative nodule size, molecular test report, type of surgery, malignancy, and aggressiveness were examined using the Wilcoxon, the chi-squared, and Fisher’s tests. Logistic regression adjusted for age and sex, with α set at .05, determined statistical significance.

## Results

### Pilot Project Patients’ Baseline Characteristics

A total of 314 patients qualified for the pilot project over a 16 month period, with 207 (65.9%) having Bethesda III nodules and 107 (34.1%) having Bethesda IV nodules. Molecular testing yielded a result of negative in 238 (75.8%) cases and positive in 76 (24.2%) cases. The mean age for positive patients was 51.77 (SD = 14.34), while that for negative patients was 54.38 (SD = 13.41; *P* = .2198). Across both phases, a congruent female to male gender distribution of 14:3 was reported (*P* = .9138). Positive patients and negative patients had a mean tumor size on ultrasound of 2.4 cm (SD = 0.84) and 2.07 cm (SD = 0.73), respectively (*P* = .0025). Among positive patients, 35 (46.1%) had a Bethesda III nodule and 41 (53.9%) had a Bethesda IV nodule. Conversely, among negative patients, 172 (72.3%) had a Bethesda III nodule, and 66 (27.7%) had a Bethesda IV nodule (*P* < .0001).

Of patients with a positive molecular test report, 67 (88.2%) underwent surgery, 5 (6.6%) were referred to surgeons outside of our institution, and 4 patients (5.3%) refused surgery. All patients, but 1 (0.4%), with a negative molecular test report proceeded with routine surveillance. This remaining negative patient received a benign histopathological diagnosis upon surgical excision of the nodule.

### Pre- and Post-Pilot Project Surgical Patients’ Baseline Characteristics

In the post-pilot project surgical group, which spanned 10 months, 67 patients were included. The pre-pilot project surgical group, which was time-matched as well for 10 months, included 71 patients. The mean age of patients in the pre-pilot project surgical group was 54.56 years (SD = 14.44), whereas in the post-pilot project surgical group, it was slightly lower at 52.82 years (SD = 14.21; *P* = .4727). Additionally, when assessing tumor size as measured by ultrasound, the mean tumor size was 2.34 cm (SD = 0.94) in the pre-pilot project surgical group and 2.31 cm (SD = 0.80) in the post-pilot project surgical group (*P* = .9306). Female-to-male ratios remained similar across both groups with a *P* value of .2532. In the pre-pilot project surgical group, 48 patients (67.6%) had a Bethesda score of III and 23 patients (32.4%) had a Bethesda score of IV. In the post-pilot project surgical group, 31 patients (46.3%) had a Bethesda score of III and 36 patients (53.7%) had a Bethesda score of IV (*P* = .0113). Baseline characteristics are recapitulated in Table 1.

**Table 1.** Baseline Characteristics for Pre- and Post-Pilot Project Surgical Patients.

Variable	Pre-pilot project		Post-pilot project		*P* value
	n = 71	%	n = 67	%	
Age, mean (SD)	54.56	14.44	52.82	14.21	0.4727
Tumor size (cm), mean (SD)	2.34	0.94	2.31	0.80	0.9306
Gender					
Male	16	22.5	10	14.9	0.2532
Female	55	77.5	57	85.1	
Bethesda score					
III	48	67.6	31	46.3	0.0113
IV	23	32.4	36	53.7	

### Surgical Outcomes of Pre- and Post-Pilot Project Surgical Patients

**Figure 1.**
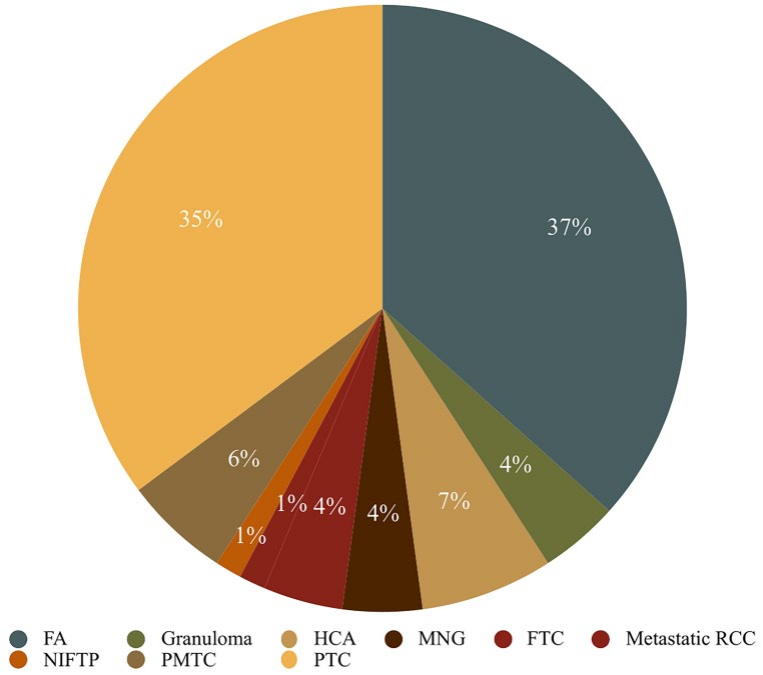
Histopathological diagnoses for pre-pilot project surgical patients. FA, follicular adenoma; FTC, follicular thyroid carcinoma; HCA, Hürthle cell adenoma; MNG, multinodular goiter; NIFTP, noninvasive follicular thyroid neoplasm with papillary-like nuclear features; PMTC, papillary thyroid microcarcinoma; PTC, papillary thyroid carcinoma; RCC, renal cell carcinoma.

**Figure 2.**
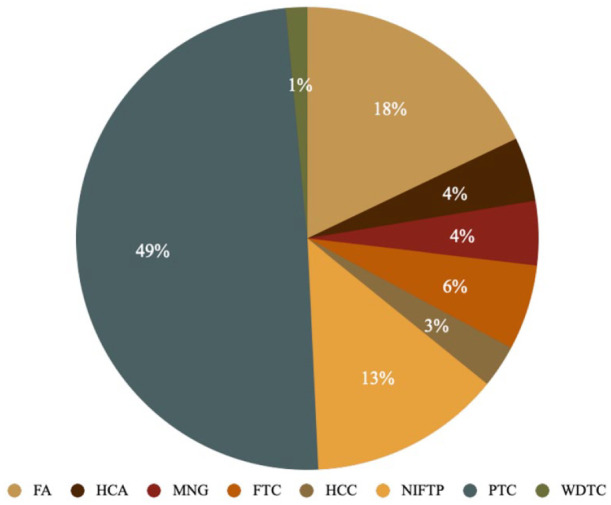
Histopathological diagnoses for post-pilot project surgical patients. FA, follicular adenoma; FTC, follicular thyroid carcinoma; HCA, Hürthle cell adenoma; HCC, Hürthle cell carcinoma; MNG, multinodular goiter; NIFTP, noninvasive follicular thyroid neoplasm with papillary-like nuclear features; PTC, papillary thyroid carcinoma; WDTC, well differentiated thyroid carcinoma.

Postoperative pathology reports revealed a 47.9% surgical malignancy/NIFTP rate in the pre-pilot project surgical group, with a distribution of 33 malignant tumors, 1 NIFTP, and 37 benign ones (Figure 1). The post-pilot project surgical project group had 40 malignant histopathologies, 9 NIFTPs, and 18 benign ones (Figure 2), exhibiting a statistically significantly-higher surgical malignancy/NIFTP rate of 73.1% (*P* = .0025). Univariate logistic regression showed an odds ratio of 2.962 (95% CI: 1.45-6.05, *P* = .0028) when comparing the post-pilot project surgical group to the pre-pilot project surgical group in terms of tumor malignancy. After adjusting for age and sex, multivariate logistic regression demonstrated an akin odds ratio of 2.988 (95% CI: 1.45-6.17, *P* = .0031) for the aforementioned comparison.

The pre-pilot project surgical group harbored a cancer aggressiveness rate of 23.5%, while that of the post-pilot project surgical group was 14.3% (*P* = .2818). The distribution of aggressive features across both groups is delineated in Table 2. In the univariate model, the odds ratio for cancer aggressiveness for the post-pilot project surgical group in comparison with the pre-pilot project surgical group is 0.542 (95% CI: 0.18-1.67, *P* = .2859). The multivariate model, which accounts for age and sex, reveals a similar odds ratio of 0.580 (95% CI: 0.19-1.82, *P* = .3502) for the same comparison parameters.

**Table 2.** Histopathological Aggressive Features for Pre- and Post-Pilot Project Malignant/NIFTP Patients.

Aggressive feature	Pre-pilot project	Post-pilot project
	n = 8 (out of 34)	n = 7^a^ (out of 49)
ETE	0	2
LNM	5	3
High-risk PTC features		
Solid/trabecular	1	2
Tall cell	1	1
Other	1	0
Metastatic RCC		

Abbreviations: ETE, extrathyroidal extension; LNM, lymph node metastasis; NIFTP, noninvasive follicular thyroid neoplasm with papillary-like nuclear features; PTC, papillary thyroid carcinoma; RCC, renal cell carcinoma.

a One patient had concurrent ETE and LNM.

### Appropriateness of Surgery for Pre- and Post-Pilot Project Surgical Patients

**Figure 3.**
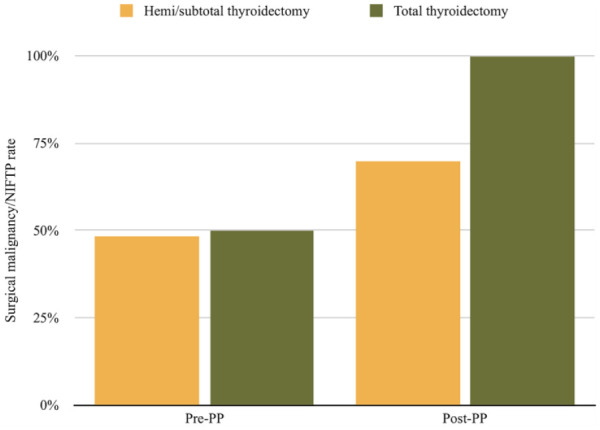
Surgical malignancy/NIFTP rates across both groups based on the type of surgical intervention. NIFTP, noninvasive follicular thyroid neoplasm with papillary-like nuclear features.

In the pre-pilot project surgical group, 64 patients (90.1%) underwent a hemi/subtotal thyroidectomy, 6 (8.5%) underwent a total thyroidectomy, and 1 (1.4%) underwent a completion thyroidectomy. The post-pilot project surgical group comprised 60 (89.6%) hemi/subtotal thyroidectomy patients and 7 (10.4%) total thyroidectomy patients. When stratified, the surgical malignancy/NIFTP rate for hemi/subtotal thyroidectomy patients was 48.4% and 50% for total thyroidectomy patients in the pre-pilot project surgical group (*P* = 1; Figure 3). Whereas, the surgical malignancy/NIFTP rates for patients undergoing hemi/subtotal and total thyroidectomies in the post-pilot project surgical group were much higher at 70% and 100%, respectively (*P* = .1763) as seen in Figure 3. In terms of cancer aggressiveness, the pre-pilot project surgical group conferred a rate of 22.6% for hemi/subtotal thyroidectomy patients and 33.3% for total thyroidectomy patients (*P* = 1). For the post-pilot project surgical group, cancer aggressiveness rates were 7.1% and 57.1% for patients undergoing hemi/subtotal and total thyroidectomies, respectively, demonstrating a statistically-significant difference (*P* = .0049).

## Discussion

The ThyroSeqv3 molecular test pilot project was implemented in light of the exacerbated health inequities brought to the forefront during the COVID-19 pandemic. Its objective was to democratize access to sophisticated, efficient, and reliable diagnostic technology, ensuring that all patients, particularly in a country with a single-payer health care system, benefit from equitable and timely access to cutting-edge care.^8^ The rationale was simple—the alleviation of uncertainty surrounding one’s diagnosis and prognosis should not solely be available to those with financial means to pay out-of-pocket for molecular testing. With a wealth of recent research enhancing our understanding of molecular profiling and its role in risk stratification, the launch of this pilot project is particularly well-timed. It aims to integrate this expanding knowledge into assessing clinical practicality and effectiveness of the test, setting the stage for what could be a transformative, long-term implementation across Québec’s health care landscape. This project not only aligns with latest scientific insights but also positions Québec to potentially set a precedent for the broader implementation of such advanced molecular diagnostics across Canada.

Our study’s findings revealed that throughout a 16 month duration, the pilot project facilitated provision of publicly-funded molecular testing to 314 patients with CITNs. This in-office diagnostic tool, while providing patients with a greater understanding of their pathology, has also effectively aided health care professionals in the decision-making process regarding further management. The 2015 ATA guidelines stipulate that the primary use of molecular markers in CITNs is for ruling in vs. ruling out malignancy, with an ancillary utility in surgical decision-making. It further states that an ideal “rule-in” test should have a positive predictive value (PPV) for histopathologically-proven malignancy similar to a malignant cytologic diagnosis (98.6%), and an ideal “rule-out” test should have a negative predictive value (NPV) similar to a benign cytologic diagnosis (96.3%).^15^ Of all patients part of the pilot project, 75.8% received a negative molecular testing report and thereby successfully avoided the traditional next step of a diagnostic hemithyroidectomy in the majority of patients. Steward et al’s prospective, blinded, multicenter cohort study concerning CITNs demonstrated ThyroSeqv3’s NPV to be 97% (95% CI: 93%-99%), surpassing the threshold set out by ATA to qualify as an ideal rule-out test.^8^ Similarly, a 2021 systematic review and meta-analysis analyzing ThyroSeqv3 diagnostic capability in CITNs revealed a 96% NPV (95% CI: 83%-88%), while a retrospective study published a year earlier revealed a NPV of 98.3% (95% CI: 96.1%-99.3%) for CITNs.^16,17^

When assessing outcomes of surgical patients before and after the implementation of pilot project, the distribution of cytological diagnoses of thyroid nodules that subsequently underwent surgical resection across the 2 groups varied inversely: The pre-pilot project surgical group harbored a higher proportion of Bethesda III nodules (67.6%) in comparison with Bethesda IV nodules (32.4%), while the post-pilot project surgical group had a higher Bethesda IV (53.7%) to Bethesda III (46.3%) ratio. Bethesda III nodules are classically quite challenging to manage, as observation, repeating FNA biopsy and performing diagnostic surgery are all appropriate options. The statistically-significant shift in the ratio of Bethesda III to IV nodules undergoing surgery, particularly within the post-pilot project group where 65.9% of the patients had Bethesda III nodules, speaks to our improved ability to manage such nodules based on this personalized diagnostic test, without overtreating. This change in clinical management suggests that publicly-funded molecular testing enhanced diagnostic accuracy for CITNs and may have prevented futile surgeries for lower risk nodules, while appropriately selecting patients with higher risk nodules for surgery.

Following the introduction of the pilot project, there was a statistically-significant increase in the surgical malignancy/NIFTP rate, rising from 47.9% to 73.1%. A univariate logistic regression analysis yielded an odds ratio of 2.962, indicating that patients who underwent publicly-funded ThyroSeqv3 molecular testing followed by surgical resection had almost a 3-fold increase in likelihood of surgery being warranted compared with those without access to this advanced and costly diagnostic technology. This suggests that the pilot project facilitated a more targeted and precise selection of cases for surgical intervention. Upon adjustment for age and gender variables, the odds ratio escalated slightly to 2.988. Our study’s surgical malignancy/NIFTP rates are higher than those reported in existing assay validation studies conducted in a private health care setting. The surgical malignancy/NIFTP rate serves as a proxy for the test’s PPV in that only patients with a positive molecular testing report, with the exception of 1 case, underwent surgery, which has been reported to be of 66% (95% CI: 50%-77%) and 64% (95% CI: 51%-77%) for CITNs.^18,19^ Our study’s increased PPV of 73.1% may be ascribed to the stricter, evidence-based inclusion criteria employed. TIRADS 3 nodules are considered “mildly suspicious,” while TIRADS 4 nodules are “moderately suspicious.”^20^ By restricting the sample to TIRADS 3 and 4, nodules are more likely to be malignant, naturally leading to a higher PPV because the pretest probability of malignancy is already somewhat elevated in this patient cohort. Furthermore, concentrating on thyroid nodules ranging from 1 to 4 cm in size may be indicative of selecting patients with an intermediate likelihood of malignancy.^21^ This size range is also often the target for diagnostic evaluations and surgery, which could lead to a higher PPV if the nodules are appropriately selected for these procedures. This judicious approach to patient selection exemplifies optimal utilization of public health resources and economic stewardship. With respect to the observed rates of cancer aggressiveness, the pre-pilot project surgical phase had a higher rate of 23.5%, in contrast to the post-pilot project surgical phase’s rate of 14.3%. This variation, while noteworthy, did not achieve statistical significance in either univariate or multivariate analysis model (*P* = .2859, *P* = .3505). This apparent discrepancy may be elucidated by the refined diagnostic acumen introduced with the pilot project. The project’s advent has enabled a more intricate understanding of CITNs, particularly in detecting genetic markers that portend a predisposition to aggressive cancer phenotypes. This advancement facilitates a layered approach to risk assessment, empowering clinicians with the capability to more precisely diagnose and categorize thyroid cancers, which are increasingly being detected at a nascent stage, when they are typically less advanced and exhibit less aggressive features.^22^

The proportion of hemi/subtotal to total thyroidectomies remained consistent across both groups, yet the appropriateness of surgery demonstrated significant variability. The surgical malignancy/NIFTP rate for hemi/subtotal thyroidectomies increased from 48.4% to 70%, while total thyroidectomies saw an impressive increase from 50% to 100%. This data shift implies that with the help of the pilot project, all patients that underwent a total thyroidectomy, which necessitates lifelong follow-up and medication for the patient, as well as being resource-intensive for the health care system, indeed had malignant disease. The impeccable success rate exemplifies the profound impact of publicly-funded ThyroSeqv3 molecular testing in refining surgical judgment. In evaluating suitability of thyroid surgery, it is critical to consider the prevalence of aggressive cancers. The 2015 ATA guidelines recommend a total thyroidectomy for patients with indeterminate nodules that are cytologically suspicious for malignancy, positive for known mutations specific for carcinoma, sonographically suspicious, large (>4 cm), or in patients with familial thyroid carcinoma or history of radiation exposure.^15^ When encountering CITNs ranging from 1 to 4 cm with a TIRADS score of 3 or 4, and absent familial or personal medical history, the decisive factor for proceeding with a total thyroidectomy becomes the mutational profile solely. Our study observed no statistically-significant variance in the rates of cancer aggressiveness between patients undergoing hemi/subtotal versus total thyroidectomy in the pre-pilot project surgical group. Conversely, in the post-pilot project surgical group, a statistically-significant increase in cancer aggressiveness (*P* = .0049) was documented—from 7.1% in hemi/subtotal thyroidectomy patients to 57.1% in those who had a total thyroidectomy, underscoring the pilot project’s effectiveness in appropriately guiding the extent of thyroidectomy due to preoperative insights on aggressive malignancies. This emphasizes the potential of molecular testing to streamline therapeutic strategies, enhancing the surgical decision-making process to benefit the patient’s health and well-being.

Our investigation elucidates crucial insights regarding the ThyroSeqv3 molecular test pilot project’s utility in surgical management of CITNs at our institution. Nevertheless, it is imperative to acknowledge the inherent constraints accompanying the study. As a retrospective analysis, it is bounded by typical limitations characteristic of such methodologies. Furthermore, our study excluded patients who, in pre-pilot project phase, personally financed their molecular testing. This decision, while bolstering representation of the public health care sector, may not encompass the comprehensive scope of Canada’s health care system. Patients who opted for private molecular testing represent a distinct subgroup with potentially-different socioeconomic profiles and health care decision-making patterns. This dichotomy underscores the potential bias introduced by excluding these patients from our analysis, making it difficult to determine their representation relative to the pilot project cohort. This gap introduces a potential confounder, as it precludes a meaningful analysis of outcome differences between privately-funded patients and those within the publicly-funded system. However, their exclusion was necessary to align our methodology with the study’s primary aim—assessing the impact of democratized, publicly-funded ThyroSeqv3 testing. Additionally, the timeline of our study coincided with the undulating landscape of COVID-19-related health care restrictions. These fluctuations, manifesting in varied access to primary care, specialist consultations, and surgical wait times, may have impacted the findings, disproportionately across the 2 phases. Nevertheless, the compelling nature of our findings cannot be understated. The judicious use of molecular testing, as demonstrated by the pilot project, is poised to enhance patient prognoses significantly while concurrently alleviating the operational burden on public health care systems. The critical importance of our pilot project lies in its contribution to the precision of surgical decision-making, which directly correlates with improved patient prognoses, a reduction in unnecessary diagnostic hemithyroidectomies, and consequent fiscal benefits.

## Conclusions

The principal objective of this study was to illuminate the pilot project’s efficacy and potential applicability across the diverse provinces and territories within the Canadian Medicare framework. The ThyroSeqv3 molecular test pilot project has marked a pivotal advancement in democratizing access to cutting-edge diagnostic care, demonstrating a commendable increase in the precision of surgical decision-making for patients with CITNs. Our study has revealed that the pilot project not only reduced unnecessary surgeries by leveraging a high NPV but also ensured that surgical interventions were more judiciously allocated, evidenced by an increased surgical malignancy/NIFTP rate post-implementation. The outcomes suggest that publicly-funded molecular testing could serve as an integral component of the Canadian single-payer health care system, optimizing patient outcomes while promoting economic efficiency. This initiative sets a progressive precedent for the broader public adoption of advanced molecular diagnostics within Canadian health care.
